# Suk-SaiYasna Remedy, a Traditional Thai Medicine, Mitigates Stress-Induced Cognitive Impairment via Keap1-Nrf2 Pathway

**DOI:** 10.3390/ijms26115388

**Published:** 2025-06-04

**Authors:** Wuttipong Masraksa, Supawadee Daodee, Orawan Monthakantirat, Chantana Boonyarat, Charinya Khamphukdee, Pakakrong Kwankhao, Abdulwaris Mading, Poowanarth Muenhong, Juthamart Maneenet, Suresh Awale, Kinzo Matsumoto, Yaowared Chulikhit

**Affiliations:** 1 Graduate School of Pharmaceutical Sciences, Khon Kaen University, Khon Kaen 40002, Thailand; wuttipongmas@kkumail.com (W.M.); abdulwaris.m@kkumail.com (A.M.); 2 Division of Pharmaceutical Chemistry, Faculty of Pharmaceutical Sciences, Khon Kaen University, Khon Kaen 40002, Thailand; csupawad@kku.ac.th (S.D.); oramon@kku.ac.th (O.M.); chaboo@kku.ac.th (C.B.); poowme@kku.ac.th (P.M.); 3 Division of Pharmacognosy and Toxicology, Faculty of Pharmaceutical Sciences, Khon Kaen University, Khon Kaen 40002, Thailand; charkh@kku.ac.th; 4 Center of Evidence-Based for Traditional and Herbal Medicine, Chao Phya Abhaibhubejhr Hospital, Prachinburi 25000, Thailand; pakakrong2@gmail.com; 5 Natural Drug Discovery Laboratory, Institute of Natural Medicine, University of Toyama, 2630 Sugitani, Toyama 930-0194, Japan; juthamart_pp@hotmail.com (J.M.); suresh@inm.u-toyama.ac.jp (S.A.); 6 Graduate School of Pharmaceutical Sciences, Daiichi University of Pharmacy, Fukuoka 815-8511, Japan; k-matsumoto@daiichi-cps.ac.jp; 7 Division of Medicinal Pharmacology, Institute of Natural Medicine, University of Toyama, 2630 Sugitani, Toyama 930-0194, Japan

**Keywords:** Suk-SaiYasna, cognitive impairment, *Cannabis sativa*, cannabinoid, chronic stress, Nrf2/Keap1, neuroprotection

## Abstract

Suk-SaiYasna (SSY) is a well-documented traditional Thai herbal formula in the Royal Scripture of King Narai’s Traditional Medicine. SSY contains *Cannabis sativa* leaves as a key ingredient and has traditionally been used to promote sleep, alleviate stress-related symptoms, and stimulate appetite. This study aimed to investigate the neuroprotective effects of SSY in a mouse model of unpredictable chronic mild stress (UCMS)-induced cognitive impairment and explore the underlying mechanisms, particularly antioxidant enzyme pathways. Behavioral tests, including the Y-maze test, novel object recognition test, and Morris water maze test, demonstrated that UCMS-exposed mice exhibited cognitive impairment compared to non-stress mice. However, SSY treatment significantly improved learning and memory performance in UCMS-exposed mice. Mechanistic studies revealed that SSY reduced lipid peroxidation in the hippocampus and frontal cortex, key brain regions affected by chronic stress. Furthermore, UCMS significantly reduced the activity of antioxidant enzymes superoxide dismutase (SOD) and catalase (CAT), whereas SSY treatment restored their activity, indicating antioxidative and neuroprotective effects in vivo. Gene expression analysis further revealed that SSY regulates oxidative stress via the Nrf2/Keap1 signaling pathway. In vitro studies using 2,2′-azinobis-(3-ethylbenzothiazoline-6-sulfonic acid (ABTS) and 2,2-diphenyl-1-picrylhydrazyl (DPPH) assay confirmed the radical scavenging activities of SSY and its herbal components, demonstrating significant antioxidant potential. Phytochemical analysis identified delta-9-tetrahydrocannabinol, delta-9-tetrahydrocannabinolic acid A, and cannabinoids as bioactive compounds in SSY, along with potent antioxidants such as gallic acid, myricetin, myristicin, piperine, costunolide, and gingerol. These findings suggest that the SSY formula mitigates UCMS-induced cognitive function through its antioxidant properties via multiple pathways, including radical scavenging activities, modulating the Nrf2-Keap1 pathway, inducing the expression of HO-1, NQO1 mRNAs, and other antioxidant enzymes. This work bridges traditional Thai medicine with modern neuropharmacology.

## 1. Introduction

Chronic stress exposure induces prolonged cortisol elevation and subsequent metabolic disturbances by dysregulating the hypothalamic–pituitary–adrenal (HPA) axis [1]. This dysregulation disrupts cellular homeostasis and produces excessive reactive oxygen species (ROS) through mitochondrial dysfunction, neuroinflammation, and glutamate excitotoxicity. Furthermore, it inhibits the nuclear factor erythroid 2-related factor 2 (Nrf2)/Kelch-like ECH-associated protein 1 (Keap1) signaling cascade, which serves an essential function in cellular defense mechanisms against both xenobiotic compounds and oxidative injury through the modulation of antioxidant enzyme activity, including superoxide dismutase (SOD) and catalase (CAT) [2]. These processes contribute to neurodegeneration, particularly in the brain, an organ highly vulnerable to oxidative stress [3].

The endocannabinoid system (ECS) has recently emerged as a promising therapeutic target for emotional and cognitive deficits associated with stress. Under normal conditions, the ECS is crucial in maintaining homeostasis in response to stress [4]. Cannabinoid 1 (CB1) receptors, primarily located in the brain, regulate neurotransmitter release and modulate HPA axis activity, thereby controlling stress hormone levels. Upon the detection of stress exposure, endocannabinoids act as retrograde messengers, inhibiting excessive neurotransmitter release and dampening HPA axis hyperactivation. This prevents prolonged stress responses and reduces cortisol secretion. Furthermore, accumulating evidence suggests that cannabidiol (CBD), delta-8-tetrahydrocannabinol (delta-8-THC), delta-9-tetrahydrocannabinol (delta-9-THC), and delta-9-tetrahydrocannabinol acid (THCA-A) exert antioxidant effects by targeting the Nrf2/Keap1 pathway and CB1/CB2 receptors [5,6].

**Figure 1:**
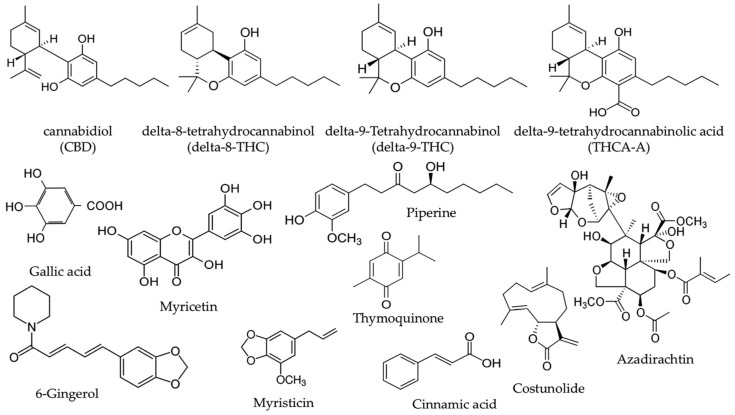
Structures of cannabidiol (CBD), delta-8-tetrahydrocannabinol (delta-8-THC), delta-9-tetrahydrocannabinol (delta-9-THC), delta-9-tetrahydrocannabinol acid (THCA-A), gallic acid, myricetin, cinnamic acid, azadirachtin, 6-gingerol, thymoquinone, piperine, myristicin, and costunolide.

The Suk-SaiYasna (SSY) formula is a traditional Thai medicine (TTM) documented in the Royal Scripture of King Narai’s Traditional Medicine. SSY is officially endorsed by the Department of Thai Traditional and Alternative Medicine and the Thai Traditional Medical Council for conventional indication as promoting sleep and stimulating appetite [7]. Recent investigations suggest that SSY may augment the efficacy of pharmacological agents aimed at γ–aminobutyric acid type A (GABAA) receptors, consequently affecting sleep and sedation results [8]. SSY formulation consists of 12 medicinal plants, including *Cannabis sativa* leaves, long pepper, neem leaves, nutmeg, clove, black cumin, costus root, magnolia flower, black pepper, dried ginger, and camphor, as outlined in Table 1 [7]. *Cannabis sativa* leaves (*Cannabis sativa* L.) constitute the most abundant ingredient, comprising 15.38% of the formulation. Among the bioactive compounds in *Cannabis sativa*, CBD, delta-8-THC, delta-9-THC, and THCA-A are the primary active constituents. In addition, other active compounds from the various herbal components of SSY include gallic acid, myricetin, myristicin, piperine, and 6-gingerol (Figure 1), all of which exhibit potent anti-oxidative and neuroprotective properties [9,10,11,12,13].

Although the traditional applications and chemical profile of SSY suggest its potential to mitigate neuronal damages induced by chronic stress, its efficacy in alleviating stress-induced cognitive impairment, particularly concerning oxidative brain damage and impaired neurogenesis, remains unclear. Therefore, the present study aimed to investigate the effect of SSY on stress-induced cognitive impairment and elucidate its underlying molecular mechanisms.

## 2. Results

### 2.1. Effect of SSY in Antioxidant Activity by DPPH and ABTS Assay

In this study, we evaluated the antioxidant activity of extracts from 13 medicinal plants using the 2,2-diphenyl-1-picrylhydrazyl (DPPH) and 2,2′-azinobis-(3-ethylbenzothiazoline-6-sulfonic acid) (ABTS) assays. Antioxidant activity was quantified using the IC_50_ value. The results indicated that *Cinnamomum bejolghota* Buch.-Ham and *Mesua ferrea* Linn. exhibited the strongest antioxidant activity in both assays, demonstrating superior free radical scavenging ability. Among the other plants, *Piper retrofractum*, *Zingiber officinale*, *Piper nigrum*, *Aucklandia lappa*, and *Azadirachta indica* displayed notable antioxidant activity. However, their IC_50_ values were higher than those of *Mesua ferrea* and *Cinnamomum bejolghota* (Table 2).

### 2.2. Effect of SSY on UCMS-Induced Cognitive Deficits

**Figure 2:**
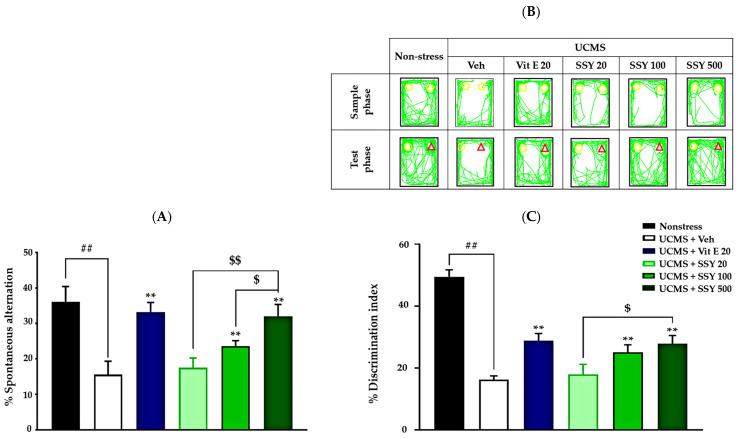
The effect of Suk-SaiYasna (SSY) and vitamin E on the UCMS-induced cognitive impairment in the Y-maze test (**A**) and NORT (**B**,**C**). Yellow circle represents the familial object and red triangle represents the novel object. Each column represents the mean ± S.E.M. (*n* = 12). ^##^ *p* < 0.001 compared to the vehicle-treated non-stress group. ** *p* < 0.001 compared to the vehicle-treated UCMS group. ^$^ *p* < 0.05, ^$$^ *p* < 0.001 compared with different doses of the SSY.

The Y-maze test and novel object recognition test (NORT) were used to assess spatial and non-spatial working memory, respectively, in UCMS-exposed animals. In the Y-maze test, UCMS-exposed mice exhibited cognitive impairment, as indicated by a significant reduction in spontaneous alternation compared to the non-stress group. However, UCMS-exposed mice treated with vitamin E or SSY demonstrated significantly improved spontaneous alternation in a dose-dependent manner compared to the vehicle-treated UCMS group (Figure 2A).

The NORT also revealed cognitive deficits in UCMS-exposed mice. During the training phase, when two identical objects were presented, no significant differences in exploration time were observed. In the test phase, the non-stressed group spent significantly more time exploring the novel object than the familiar one. In contrast, vehicle-treated UCMS mice failed to distinguish between the two objects, indicating UCMS-induced impairment in non-spatial working memory. Notably, UCMS-exposed mice treated with vitamin E or SSY (100 or 500 mg/kg/day for three weeks) exhibited significantly improved discrimination performance in this test (Figure 2B,C). These findings suggest that vitamin E and SSY have therapeutic potential for mitigating UCMS-induced cognitive impairments.

**Figure 3:**
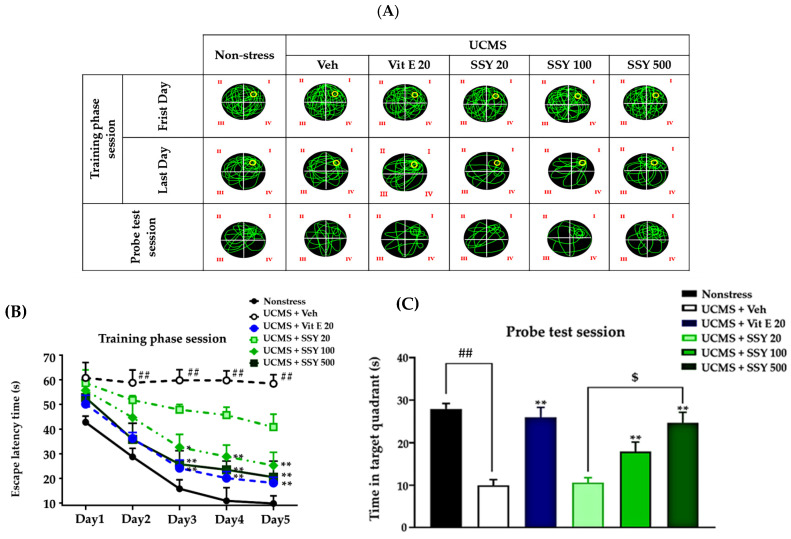
The effect of Suk-SaiYasna (SSY) and vitamin E on the UCMS-induced cognitive impairment in the Morris water maze (MWM) (**A**), training phase session (**B**), and probe test session (**C**). Yellow circle represents the hidden platform. Each column represents the mean ± S.E.M. (*n* = 12). ^##^ *p* < 0.001 compared to the vehicle-treated non-stress group. * *p* < 0.05, ** *p* < 0.01 compared to the vehicle-treated UCMS group. ^$^ *p* < 0.05 compared with different doses of the SSY.

The Morris water maze (MWM) was used to assess spatial reference memory (Figure 3A). All groups attained the platform location, evidenced by reduced escape latency, showing a certain level of learning capability. The vehicle-treated UCMS-exposed mice exhibited significantly longer escape latencies during training and spent less time swimming time in the target quadrant during the probe test compared to non-stressed animals. These results indicate that UCMS exposure impairs both the acquisition and retrieval of reference memory. The repeated treatment with vitamin E and SSY greatly ameliorated these deficits in UCMS-exposed mice, as shown in Figure 3.

### 2.3. Effect of SSY on Lipid Peroxidation and the Activities of SOD and CAT in UCMS-Exposed Mouse Brain

**Figure 4:**
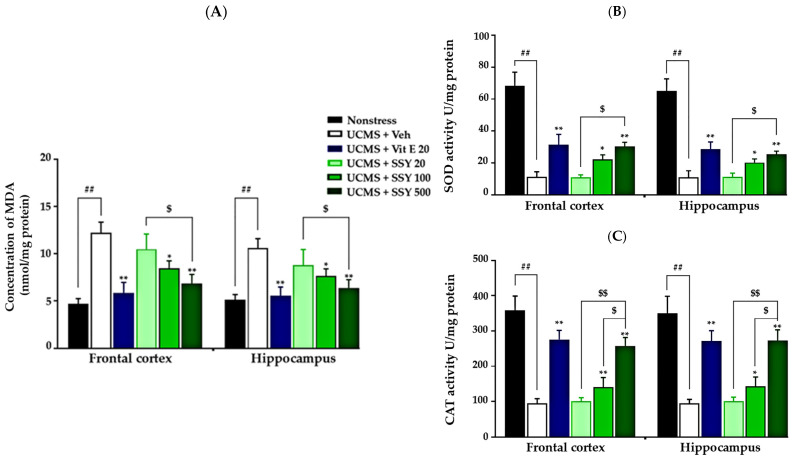
The effect of Suk-SaiYasna (SSY) and vitamin E on the UCMS-induced brain oxidative stress, lipid peroxidation assay (**A**), SOD activity (**B**), and CAT activity (**C**). Each column represents the mean ± S.E.M (*n* = 5). ^##^ *p* < 0.001 compared to the vehicle-treated non-stress group. * *p* < 0.05, ** *p* < 0.001 compared to the vehicle-treated UCMS group. ^$^ *p* < 0.05, ^$$^ *p* < 0.001 compared with different doses of the SSY.

Chronic stress-induced lipid peroxidation leads to excessive oxidative damage in the brain, contributing to neuronal dysfunction and neurodegeneration. In the present study, UCMS-exposed animals exhibited significantly increased MDA levels in the frontal cortex (FC) and hippocampus (HP) compared to non-stressed mice, indicating enhanced lipid peroxidation due to UCMS exposure. However, treatment with vitamin E or SSY (100 and 500 mg/kg/day) significantly attenuated these increases in MDA levels in both brain regions (Figure 4A).

Additionally, this study examined the effects of SSY on the activities of two key antioxidant enzymes, superoxide dismutase (SOD) and catalase (CAT), in the FC and HP of UCMS-exposed mice. As shown in Figure 4, UCMS exposure significantly reduced the activities of both enzymes compared to the non-stressed group. However, administration of vitamin E or SSY at 100 and 500 mg/kg/day significantly restored their levels (Figure 4B,C), suggesting a protective effect against UCMS-induced oxidative stress.

### 2.4. Effects of SSY on the Nrf2-Keap1 Pathway in UCMS-Exposed Mice

**Figure 5:**
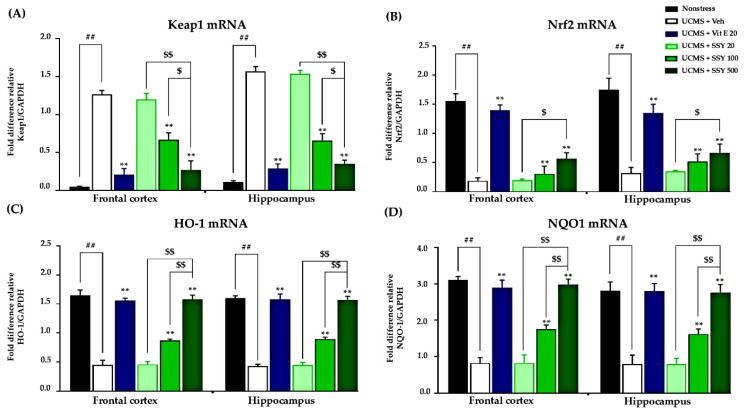
The effect of Suk-SaiYasna (SSY) and vitamin E on mRNA expressions of Keap1 (**A**), Nrf2 (**B**), HO-1 (**C**), and NQO1 (**D**) in the UCMS mouse model. Each column represents the mean ± standard error of the mean (*n* = 5). ^##^ *p* < 0.001 compared to the vehicle-treated non-stress group. ** *p* < 0.001 compared to the vehicle-treated UCMS group. ^$^ *p* < 0.05, ^$$^ *p* < 0.001 compared with different doses of the SSY.

UCMS induction resulted in significant suppression of Nrf2 gene transcription, concurrent with enhanced Keap1 mRNA levels, consequently compromising cellular antioxidant protective systems and intensifying brain oxidative stress. These molecular changes were associated with cognitive decline and neuronal oxidative damage in UCMS-exposed animals. However, daily treatment with vitamin E or SSY significantly reversed these alterations, restoring Nrf2 and Keap1 expression levels and suggesting a reduction in the inhibition of the Nrf2-Keap1 pathway in treated UCMS-exposed mice (Figure 5A,B). To validate these findings, we examined the expression levels of downstream effectors of Nrf2, specifically heme oxygenase-1 (HO-1) and NAD(P)H quinone dehydrogenase 1 (NQO1) (Figure 5C,D). Our findings indicate that SSY significantly attenuates brain oxidative stress induced by UCMS through regulation of the Nrf2-Keap1 signaling pathway, consequently providing neuroprotection and potentially ameliorating stress-related cognitive impairment.

### 2.5. LC-MS/MS Analysis of the Constituents of the SSY and Cannabis sativa Linn. Extract

**Figure 6:**
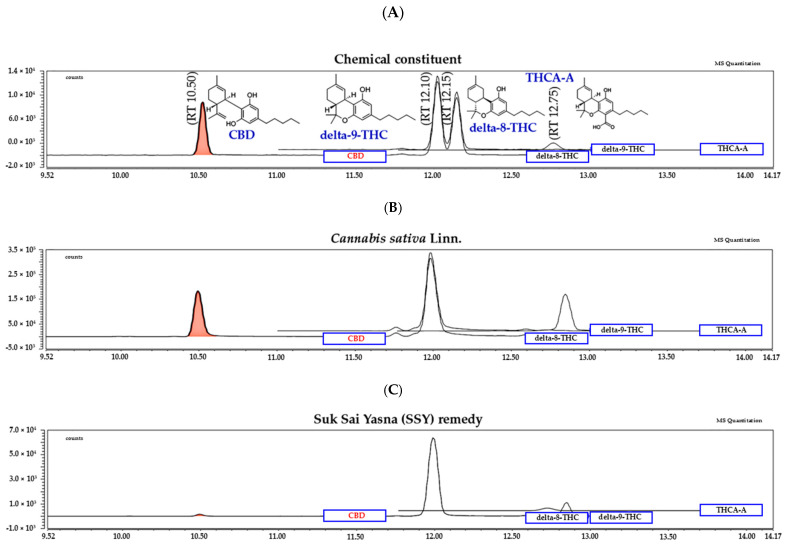
Chromatogram of chemical constituents CBD (10.50), delta-9-THC (12.10), delta-8-THC (12.15), and THCA-A (12.75), respectively (**A**), *Cannabis sativa* Linn. (**B**), and the Suk-SaiYasna (SSY) remedy (**C**).

To characterize the chemical composition of SSY and *Cannabis sativa* Linn., we performed LC-MS/MS analysis on the 95% ethanolic extract of SSY and the 95% ethanolic extract of *C. sativa* leaves using standard references, including cannabidiol (CBD), delta-9-tetrahydrocannabinol (Δ^9^-THC), delta-8-tetrahydrocannabinol (Δ^8^-THC), and tetrahydrocannabinolic acid A (THCA-A). The retention times (RTs) for these compounds were 10.50, 12.10, 12.15, and 12.75 min, respectively (Figure 6A).

The chromatogram of the 95% ethanolic extract leaves of *Cannabis sativa* Linn. (Figure 6B), the 95% ethanolic extract of the SSY remedy (Figure 6C). The quantity of chemical constituents in the leaves of *Cannabis sativa* Linn. and Suk-SaiYasna (SSY) are shown in Table 3.

### 2.6. HPLC Analysis of the Constituents of the SSY Extracts and the Validation Method

**Figure 7:**
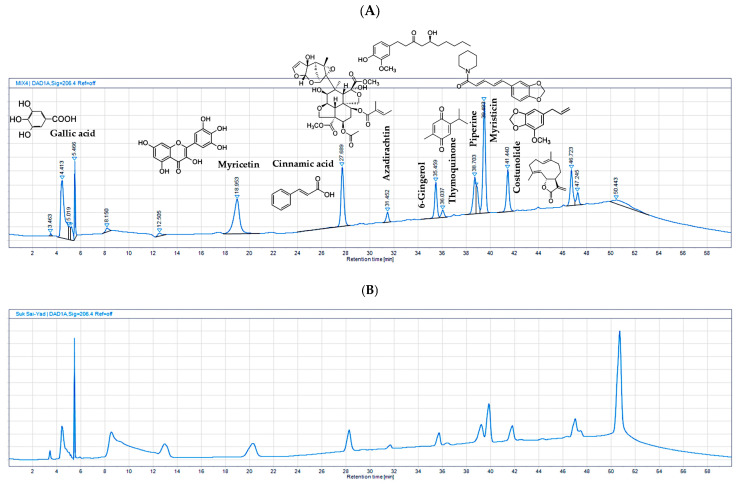
Chromatogram of chemical constituent gallic acid (5.466), myricetin (18.953), cinnamic acid (27.689), azadirachtin (31.452), 6-gingerol (35.459), thymoquinone (36.037), piperine (38.703), myristicin (39.483), and costunolide (41.440), respectively (**A**), and the Suk-SaiYasna (SSY) remedy (**B**).

Gallic acid, myricetin, cinnamic acid, azadirachtin, 6-gingerol, thymoquinone, piperine, myristicin, and costunolide served as the chemical constituents in the HPLC analysis of the 95% ethanolic extract of the SSY remedy. The method validation demonstrated the reliability and suitability of the selected method to quantify all the chemical constituents present in the SSY extract and the retention times (Figure 7).

The linearity of the method exhibited a high correlation between the peak area and concentration, with an R^2^ greater than or equal to 0.99. Accuracy, determined by the percentage recovery of spiked standards, consistently ranged between 80% and 115%. Precision, including repeatability and intermediate precision, demonstrated percentage relative standard deviations (%RSDs) of less than 6%. The limit of detection (LOD) and limit of quantitation (LOQ) were established with signal-to-noise (S/N) ratios greater than or equal to 3 and 10, respectively. UV peak purity analysis in OpenLAB CDS version 2.3 showed values greater than or equal to 990.00. In the robustness testing, %RSD was less than or equal to 6%. These validation parameters collectively confirmed the reliability of the analytical method for the analysis of the nine standard references (Supplementary Materials, Table S13). Subsequently, HPLC was employed to quantify the chemical constituents in the 95% extract of the SSY remedy and single herbs, as shown in Table 4. The HPLC analysis of the 95% extract medicinal powder of SSY revealed the presence of several key chemical constituents. Among these, gallic acid was the most abundant, with a content of 167.777 mg/g in SSY. Other major constituents in SSY included myricetin (17.225 mg/g), piperine (8.853 mg/g), costunolide (7.911 mg/g), and myristicin (6.847 mg/g). These findings highlight the diverse chemical profile of SSY, with gallic acid, myricetin, piperine, myristicin, and costunolide as the dominant constituents in the extract.

## 3. Discussion

The present study demonstrated that SSY, a traditional Thai herbal formula, mitigates cognitive impairment and oxidative brain damage in a UCMS mouse model. The effects of SSY are mediated by its antioxidant and cannabinoid-related components, which contribute to both antioxidant activity and modulation of the endocannabinoid system (ECS).

To investigate the potential protective effects of SSY and vitamin E on stress-induced cognitive dysfunction, we employed the UCMS model, a well-established paradigm that mimics chronic psychological stress in humans. Chronic stress is a key risk factor for cognitive decline and is known to impair brain regions critical for learning and memory, particularly the hippocampus and prefrontal cortex [3,14]. Therefore, this model provides a relevant framework to study stress-related cognitive impairment and the neuroprotective effects of therapeutic agents. In the present study, we assessed the cognitive performance of UCMS-exposed mice using three behavioral tests: the MWM test, the Y-maze test, and the NORT. The MWM and Y-maze tests evaluate spatial reference memory and working memory, respectively, both of which depend on hippocampal function [15,16]. The NORT assesses recognition and working memory, in which the perirhinal cortex plays an important role [17,18]. Impaired performance in these tasks indicated that UCMS exposure led to cognitive deficits likely associated with dysfunction in both the hippocampus and cortical regions. Importantly, administration of SSY and vitamin E during the UCMS exposure period significantly mitigated these cognitive deficits. Based on these findings, we hypothesize that SSY and vitamin E exert neuroprotective effects by preventing UCMS-induced neuronal damage in the hippocampus (HP) and frontal cortex (FC), thereby attenuating stress-related cognitive decline.

To test this hypothesis, we analyzed the effects of SSY and vitamin E on UCMS-induced lipid peroxidation, antioxidant enzyme activity, and the mRNA expression of the Keap1-Nrf2 pathway, a key antioxidant defense mechanism, in the HP and FC. These brain regions are particularly vulnerable to chronic stress and oxidative stress [19,20]. Furthermore, oxidative stress is a major etiological factor in cognitive decline, leading to impairments in learning, memory, and decision-making through its detrimental effects on brain structure and function [19]. Our results demonstrated that UCMS exposure significantly increased lipid peroxidation and reduced SOD and CAT enzyme activities in the FC and HP. However, treatment with SSY or vitamin E effectively prevented these oxidative stress-induced changes. Therefore, it is plausible that the anti-dementia effects of SSY in UCMS-exposed mice are mediated by its ability to counteract UCMS-induced reductions in SOD and CAT activities and lipid oxidation in the FC and HP, suggesting a strong antioxidant and neuroprotective effect of SSY formula.

The proposed hypothesis is further supported by the present study, which focused on mRNA expression related to the Keap1-Nrf2 pathway, a key cellular defense mechanism against oxidative stress. In this pathway, Nrf2, which is released under oxidative stress, translocates into the nucleus and binds to antioxidant response elements (AREs), leading to the transcription of various antioxidant genes such as HO-1 and NQO-1 [21]. Both of these enzymes are phase II detoxification enzymes, which protect cells from oxidative damage and inflammation. On the other hand, Keap1 negatively regulates Nrf2 activity by inducing its degradation [21,22]. Therefore, the mRNA expression levels of Keap1 and Nrf2 are closely associated with the activity of oxidative stress defense mechanisms. Interestingly, in this study, treatment with SSY and vitamin E dose-dependently restored the expression levels of Keap1 and Nrf2 mRNA, which were upregulated and downregulated, respectively, by UCMS exposure in the HP and FC. These results strongly suggest that SSY has the potential to activate the Keap1-Nrf2 pathway in UCMS-exposed animals and enhance antioxidant defense mechanisms. This potential of SSY was further supported by the observation that SSY treatment upregulated the expression of genes encoding HO-1 and NQO1, the downstream targets of Nrf2, in the brain. Collectively, these findings indicate that SSY treatment confers neuroprotection through the amelioration of cerebral oxidative stress and enhancement of antioxidant enzymatic activity, with this mechanism being linked to modulation of the Nrf2/Keap1 signaling pathway.

To further substantiate these findings, we assessed the in vitro antioxidant capacity of SSY and its constituent herbal extracts using DPPH and ABTS radical scavenging assays. SSY demonstrated considerable antioxidant potential, with IC_50_ values of 0.682 ± 0.010 mg/mL (DPPH) and 0.199 ± 0.004 mg/mL (ABTS), indicating potent free radical scavenging activity. Interestingly, several individual herbal components in SSY exhibited particularly strong antioxidant activity. The result highlights *Cinnamomum bejolghota* as the most potent antioxidant component in the formulation with IC_50_ values of 0.011 ± 0.000 mg/mL (DPPH) and 0.013 ± 0.000 mg/mL (ABTS), followed by *Mesua ferrea* (IC_50_ values of 0.057 ± 0.000 mg/mL (DPPH) and 0.021 ± 0.001 mg/mL (ABTS)) and *Azadirachta indica* (IC_50_ values of 0.682 ± 0.010 mg/mL (DPPH) and 0.199 ± 0.004 mg/mL (ABTS)). Their strong free radical scavenging activities suggest that these herbs may play an important role in the antioxidant capacity of SSY.

Taken together, the in vitro and in vivo findings support the hypothesis that SSY’s neuroprotective effects are mediated through a combination of direct antioxidant properties and the preservation of endogenous antioxidant enzyme activity in stress-vulnerable brain regions.

Finally, we conducted LC-MS/MS analysis to characterize the chemical composition of psychoactive compounds in the SSY remedy and *Cannabis sativa* Linn., as well as HPLC analyses of SSY to quantify its chemical constituents using reference standard chemicals as markers [23,24,25,26,27,28,29,30,31,32,33,34,35,36,37,38,39,40]. Gallic acid, myricetin, 6-gingerol, cinnamic acid, azadirachtin, thymoquinone, piperine, myristicin, and costunolide possess robust UV-absorbing moieties, rendering them very suitable for detection via HPLC with diode array detectors. In contrast, CBD, THCA-A, Δ^8^-THC, and Δ^9^-THC are cannabinoids that usually exist in smaller amounts and present analytical challenges due to their structural similarity and potential matrix interferences in plant extracts. In particular, Δ^8^-THC and Δ^9^-THC are structural isomers, differing only in the position of a double bond, as shown in Figure 1. Their very similar chemical structures make them difficult to separate and quantify reliably using HPLC alone. Therefore, LC-MS/MS was employed as it offers higher sensitivity and selectivity, as well as the ability to clearly distinguish and quantify closely related isomers based on their specific mass-to-charge (m/z) transitions. Through these analyses, we identified CBD, Δ^9^-THC, and THCA-A in the extracts of both SSY and *Cannabis sativa* Linn. A growing body of evidence suggests that the endocannabinoid system, particularly cannabinoid receptors, plays a crucial role in neuroprotection and cognitive function. Additionally, CBD and Δ^9^-THC have been reported to activate the Keap1-Nrf2 pathway through the endocannabinoid system, particularly via cannabinoid receptors [41]. These findings suggest that the cannabinoid-related constituents of SSY at least partly contribute to its mitigating effects on cognitive deficits in UCMS-exposed animals. Furthermore, as summarized in Table 4, the SSY formulation was found to contain various bioactive compounds, such as gallic acid, piperine, myricetin, and 6-gingerol. The numerous components of medicinal plants contain many active molecules from different families, including flavonoids and phenolic compounds, which are recognized for their therapeutic properties. Previous studies have demonstrated the neuroprotective effects of certain identified bioactive constituents. Gallic acid and myricetin have improved chronic restraint stress-induced cognitive deficit by preventing the neuronal loss and brain oxidative damage in the hippocampus and prefrontal cortex [42,43,44]. Research by Rinwa and colleagues demonstrated that piperine attenuated cognitive impairment and neuroinflammatory responses in a chronic unpredictable stress murine model [45], whereas separate investigations revealed that myricetin conferred neuroprotection through facilitation of Nrf2 nuclear translocation and upregulation of heme oxygenase-1 (HO-1) and NAD(P)H: quinone oxidoreductase 1 (NQO 1) expression, concurrently decreasing MDA concentrations and enhancing SOD/catalase enzymatic activities in cuprizone-treated mice [9,46]. These bioactive compounds presumably contribute to SSY’s protective effects against UCMS-induced cognitive deterioration by mitigating oxidative neuronal injury within cerebral tissues. Consequently, these findings demonstrate that SSY represents a potential therapeutic intervention for alleviating cognitive dysfunction and neurodegeneration associated with chronic stress exposure via modulation of the Keap1-Nrf2 signaling pathway and antioxidant mechanisms.

## 4. Materials and Methods

### 4.1. Plant Materials and SSY Preparation

The SSY formulation was obtained from Chao Phya Abhaibhubejhr Hospital, Prachinburi Province, Thailand. This traditional remedy is composed of twelve medicinal plants, as documented in Table 1. Taxonomic identification of the botanical materials was performed by Benjawan Leenin, Director of the Traditional Knowledge Center, Chao Phya Abhaibhubejhr Hospital Foundation. Herbarium voucher specimens were deposited and archived at the institutional repository of Chao Phya Abhaibhubejhr Hospital for future reference and verification.

All plant materials were cleaned, sectioned, dried at 50 °C, and pulverized. Components were weighed and combined according to Table 1 specifications. Individual plant powders and SSY powder underwent triple maceration with 95% EtOH at room temperature for 72 h, followed by filtration through Whatman No.1 paper. Combined extracts were concentrated via rotary evaporation under reduced pressure at 50 °C. Extraction yields were calculated on a weight-to-weight basis, and all extracts were stored at −20 °C throughout the experiment.

### 4.2. In Vitro Antioxidant Activity by DPPH and ABTS Assay [46]

The antioxidant activity of the SSY extract was evaluated using the DPPH assay, following the manufacturer’s guidelines. Specifically, 100 µL of the SSY extract was combined with 100 µL of a 0.2 mM DPPH (Sigma-Aldrich, St. Louis, MO, USA) solution. This mixture was then allowed to incubate for 30 min at room temperature. The absorbance at 550 nm was measured using a microplate reader. Trolox (Sigma-Aldrich, St. Louis, MO, USA) was the standard antioxidant. The ability to scavenge free radicals was calculated using this equation:

Percentage inhibition = (AC − AS)/AC × 100(1)

where AC is the absorbance of the control and AS is the absorbance of the sample.

The antioxidant activity of the SSY extract was also assessed using the 2,2′-azinobis-(3-ethylbenzothiazoline-6-sulfonic acid) (ABTS•+) assay. In this assay, the ABTS•+ radical cation solution was generated by mixing of 7 mM ABTS (Sigma-Aldrich, St. Louis, MO, USA) with 2.45 mM potassium persulfate in water and incubating the solution in the dark at room temperature for 12–16 h. For the assay, 50 µL of the SSY extract was combined with 100 µL of the prepared ABTS•+ solution, and the reaction was allowed to proceed for 15 min. After incubation, the absorbance was measured at 700 nm. Trolox served as the reference antioxidant in this experiment.

### 4.3. Animal

Seventy-two ICR male mice (5 weeks old, weight 20–30 g) were purchased from Nomura Siam International Company (Bangkok, Thailand). The animals were housed in the Laboratory Animal Unit at the Faculty of Pharmaceutical Sciences, Khon Kaen University. The facility was maintained at a temperature of 22 ± 2 °C with 45 ± 2% humidity under a 12 h light/dark cycle (06:00 am to 06:00 pm). Animal handling and care procedures were approved by the Khon Kaen University Animal Ethics Committee (Approval No. IACUC-KKU-103/64-01/10/2021). All protocols were strictly implemented according to the Guiding Principles for the Care and Use of Animals (NIH Publications No.80-23, revised in 2011) and complied with the ARRIVE guideline 2.0.

### 4.4. Unpredictable Chronic Mild Stress (UCMS)

**Figure 8:**
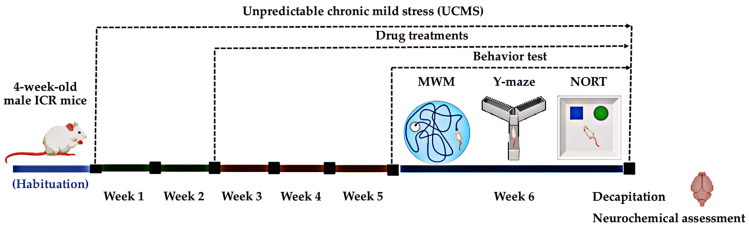
A schematic presentation of experimental protocols. Figure created with BioRender.com, accessed on 15 November 2024.

Mice were induced to demonstrate depressive-like behaviors using a UCMS model. The animals were divided into six groups at random: five groups underwent a six-week UCMS protocol (weeks 1–6), while the non-stress group, serving as the control, remained in normal housing. The UCMS protocol consisted of various stressors randomly applied throughout the light/dark cycle, repeated for six weeks. These included: 21 h in a wet cage, two 36 h periods of continuous light exposure, two periods of cat noise exposure (3 and 5 h each), two 2 h periods of paired caging, cage tilting at a 45-degree angle (twice), limited food access (5 pellets for 1 h, twice), and exposure to an empty water bottle (3 h, twice) (Figure 8). This multifaceted approach was designed to create a constant, unpredictable stress environment, reliably inducing cognitive impairment behaviors in mice (Figure 8).

### 4.5. Drug Administration

A non-stress control group received only a vehicle solution (0.5% sodium carboxymethyl cellulose, SCMC, HiMedia laboratories, Mumbai, India). The UCMS-exposed groups were administered either SCMC, vitamin E (Sigma-Aldrich, St. Louis, MO, USA, 100 mg/kg/day), or SSY powder (20, 100, or 500 mg/kg), all suspended in 0.5% SCMC. The dose of administration of SSY was derived from the clinical dose (500–2000 mg/day) prescribed in the hospital. These doses were converted into the appropriate dose for mice by the following equation: human equivalent dose (HED, mg/kg) = mouse dose (mg/kg) × mouse Km/Human Km), where Km is the correction factor [47]. Each treatment was administered daily for three weeks, starting from day 21. During behavioral testing, drugs were given one hour before the test (Figure 8).

### 4.6. Behavioral Studies

#### 4.6.1. Y-Maze Test

The Y-maze consists of three identical arms of equal size (40 cm long, 3 cm wide at the bottom, and 12 cm wide at the top) arranged at a 60° angle. This test assesses spontaneous alternation behavior. During an 8 min trial, the number and sequence of arm entries were recorded. Spontaneous alternation was defined as consecutive entries into three different arms, for example, 123, 321, 132, 213, etc., but not repeated entries like 131. The test evaluates cognitive functions, including working memory and spatial learning [48]. The percentage of alternation was calculated as follows:

% Alternation = (Number of alternations/Total arm entries 2) × 100

#### 4.6.2. Novel Object Recognition Test (NORT)

The NORT was conducted as previously described [48] to elucidate non-spatial working memory performance. The experimental apparatus comprised a square plastic arena with dimensions of 50 × 50 × 40 cm. The testing protocol was structured into three sequential phases: habituation, sample, and test phases. During the habituation phase, individual subjects were introduced to the arena environment for 10 min, 24 h before experimental assessment. In the sample phase, each mouse was placed within the arena containing two identical objects positioned at distinct locations for a 5 min duration. Exploratory behavior was quantified by measuring the time spent investigating each object through direct contact, olfactory exploration, and visual orientation.

The test phase was initiated 30 min following completion of the sample phase, wherein one familiar object was substituted with a novel stimulus. Subjects were reintroduced to the arena for 5 min, during which exploration times for both the familiar and novel objects were systematically recorded and analyzed. The arena and objects were cleaned using 70% ethanol between trials to eliminate olfactory interference. A discrimination index (DI) was calculated according to the following equation [48]:

% Discrimination index = {(TN − TF)/(TN + TF)} × 100

Here, TN and TF represent the time spent exploring novel and familiar objects, respectively, during a 5 min observation period.

#### 4.6.3. Morris Water Maze (MWM) Test

The Morris water maze (MWM) test was conducted with slight modifications to the previous protocol [49] to assess spatial reference memory. The apparatus consisted of a circular pool filled with water and a transparent escape platform submerged 1 cm below the surface. Initially, a visible platform trial was conducted with the platform raised 1 cm above the water. Mice then underwent four training trials per day for five consecutive days. In each trial, the animals were placed in the pool facing the wall, with start locations varied pseudo-randomly. The latency to reach the platform (escape latency), which remained fixed throughout training, was recorded through video tracking software (ToxTrac v2.98). Following a 10 s rest on the platform, mice were placed in a high-walled chamber for 1 min to prevent the use of external visual cues. One day after the final training session, a single 60 s probe trial was conducted without the platform. Time spent in the target quadrant (Q1) was compared with other quadrants (Q2–Q4) [49].

### 4.7. Measurement of MDA Level by the Thiobarbituric Acid Reactive Substances (TBARS) Assay

Malondialdehyde (MDA) levels, serving as an indicator of lipid peroxidation, were quantified in frontal cortex and hippocampal tissues according to established protocols [14]. Brain tissues were homogenized in ice-cold phosphate buffer (5 mM, pH 7.4) at a 1:10 tissue-to-buffer ratio using a Potter–Elvehjem homogenizer fitted with a Teflon pestle (Sigma-Aldrich, St. Louis, MO, USA).

The resulting homogenates were combined with an equal volume of 10% (*w*/*v*) trichloroacetic acid (TCA; Sigma-Aldrich, St. Louis, MO, USA) and subsequently centrifuged at 8000× *g* for 10 min at 4°C. The supernatant fraction was then incubated with 0.8% (*w*/*v*) 2-thiobarbituric acid (TBA; Sigma-Aldrich, St. Louis, MO, USA) at 100 °C for 15 min. Following a cooling period, thiobarbituric acid reactive substances (TBARS) content, representing lipid peroxidation levels, was determined spectrophotometrically at 532 nm using malondialdehyde (MDA; Sigma-Aldrich, St. Louis, MO, USA) as the reference standard. Results were expressed as nmol MDA per mg protein. Protein concentrations in brain homogenates were determined using the Bradford assay method [49].

### 4.8. Determination of Superoxide Dismutase (SOD) and Catalase (CAT) Activities

The activities of SOD and CAT in the homogenates of the FC and HP were assayed using commercially kits available from Sigma-Aldrich (St. Louis, MO, USA). The protein concentrations of the FC and HP homogenates were determined using the Bradford method. Data were normalized to their respective total protein concentrations in each samples as previously reported [49].

### 4.9. Quantitative Real-Time PCR

Total RNA from the FC and HP was extracted by using TRI-zol^®^ reagent (Invitrogen^TM^, Thermo Fisher Scientific, Waltham, MA, USA), following the manufacturer’s protocol. First-strand cDNA was synthesized with oligo(dT) primers and SuperScript III reverse transcriptase (Invitrogen^TM^, Thermo Fisher Scientific, Waltham, MA, USA). QPCR was conducted using SsoAdvanced^TM^ Universal SYBR^®^ Green Supermix (Biorad, Hercules, CA, USA). The amplification process was conducted using gene-specific PCR primer sets (Pacific Science, Bangkok, Thailand), as shown in Table 5.

### 4.10. Liquid Chromatography–Mass Spectrometry with Tandem Mass Spectrometry (LC-MS/MS) Analysis

The LC-MS/MS apparatus and conditions were employed for the analysis of the three marker components in *Cannabis sativa* Linn. and the SSY remedy. CBD, delta-9-THC, and THCA-A were tested by the Institute of Nutrition, Mahidol University (INMU), conducted with a Thermoscientific, Ultimate 3000 series UHPLC (Thermo Fisher Scientific, Waltham, MA, USA), TSQ Quantis^TM^ Triple quadrupole mass spectrometer. LC-MS/MS analysis was conducted with an Accucore RP-MS 100 × 2.1 particle size 2.6 µm. Acetonitrile with 0.1% (*v/v*) formic acid (A) and water with 0.1% (*v/v*) formic acid (B) were used as mobile phases with a gradient of 50% B (initial–5.0 min), 50–25% B (1.0–7.0 min), 25–20% B (7.0–10.0 min), 20–5% B (10–15.0 min), 5% B (15.0–16.0 min), 5–50% B (16.0–18.0 min), and 50% B (18.0–23.0 min). The flow rate was established at 0.3 mL/min, with an injection volume of 2.0 μL. We consistently maintained the samples at 5 °C. We used positive and negative ions in the multiple reaction monitoring (MRM) mode of electrospray ionization tandem mass spectrometry (ESI–MS/MS) for MS detection. Optimized analytical parameters include collision energy, cone voltage, and transition settings. Additional MS parameters were as follows: positive ions were at 4 kV, and negative ions were at 3.5 kV [53]. There were 30 arbitrary units of sheath gas, 10 arbitrary units of auxiliary gas, and 1 arbitrary unit of sweep gas. The ion transfer tube was at 325 °C and the vaporizer was at 300 °C. The cycle time was 0.8 s, the Q1 resolution was 0.7 full width at half maximum (FWHM), and the Q3 resolution was 1.2 FWHM. The collision-induced dissociation (CID) gas was at 0.5 mTorr [53].

### 4.11. High-Performance Liquid Chromatography (HPLC) Analysis and Validation of the Analytical Method

The quantity of active compounds (piperine, myricetin, azadirachtin, costunolide, myristicin, gallic acid, 6-gingerol, thymoquinone, and cinnamic acid) in SSY extract and individual medicinal plants was analyzed using the HPLC technique. A reversed-phase HPLC system with a Hypersil ODS column (Agilent Technologies Inc., Santa Clara, CA, USA; 4 × 250 mm, five μm) was used in this study. The mobile phases were 0.05% phosphoric acid in ultrapure water (Solvent A) and acetonitrile (Solvent B). Gradient elution was performed as follows: 0–14 min with 80% A, 15 min with 75% A, 20 min with 65% A, 25 min with 55% A, 30 min with 45% A, 35 min with 35% A, 40 min with 25% A, 45 min with 15% A, and 55–60 min with 80% A. The flow rate was 0.5 mL/min, the temperature was maintained at 25 ± 2 °C, and detection was performed at 206 nm. To prepare the stock solution, 20 mg of the SSY extract was dissolved in methanol to achieve a concentration of 20 mg/mL, and then it was diluted with the mobile phase to the required concentrations for quantification. Before injection into the HPLC, the extract solution was filtered using a 0.45 μm nylon syringe filter. The analytical method was validated following ICH guidelines, which were adopted on 24 March 2022. Linearity was validated by using linear regression analysis to calculate the coefficient of determination (R^2^) of the standard calibration curve (*n* = 5). The limit of detection (LOD) and limit of quantitation (LOQ) were also examined, and signal-to-noise ratios were calculated (*n* = 5). The accuracy was measured by the percentage recovery of three standard concentrations in 5 replicates. Precision (%RSD) was validated for within-day and between-day determinations (*n* = 5).

### 4.12. Statistical Analysis

All results are presented as mean ± standard error of the mean (S.E.M.). Behavioral data were analyzed using Student’s t-test for comparisons between two groups or one-way analysis of variance (ANOVA) followed by Tukey’s post hoc test for multiple group comparisons. A *p*-value of less than 0.05 was considered statistically significant. Statistical analyses were performed using SigmaStat^®^ software, version 3.1 (SYSTAT Software Inc., Richmond, CA, USA).

## 5. Conclusions

The present study provides the first scientific evidence supporting the neuroprotective potential of the traditional Thai herbal remedy **SSY,** which consists of 12 herbs with *Cannabis sativa* Linn. as the main component relieves cognitive impairment in UCMS mice. SSY significantly improved memory performance in UCMS-exposed mice and mitigated oxidative stress in the frontal cortex and hippocampus by restoring SOD and CAT activities and activating the Keap1-Nrf2 antioxidant pathway, including upregulation of Nrf2, HO-1, and NQO1. Complementary in vitro assays confirmed the strong antioxidant capacity of SSY and its top-ranking components (*Cinnamomum bejolghota*, *Mesua ferrea*, and *Azadirachta indica*), while LC-MS/MS and HPLC analyses identified key bioactive compounds, including CBD, Δ^9^-THC, and various flavonoids. Given the known role of cannabinoids in Nrf2 activation via the endocannabinoid system, these constituents likely contribute to SSY’s neuroprotective effects. Together, these multi-layered findings—ranging from behavioral to molecular and phytochemical levels, provide strong scientific support for the traditional use of SSY and highlight its therapeutic potential as a multi-target strategy for alleviating stress-induced cognitive decline. This study marks an important step toward bridging traditional Thai medicine with modern neuroscience and paves the way for future translational and clinical research.

## Figures and Tables

**Table 1:** List of medicinal plants in SSY remedy.

Scientific Name	Part Used	Proportion (%)	Voucher Specimen
*Cannabis sativa* Linn.	leaves	15.385	ABH-SSY1
*Piper retrofractum* Vahl.	fruits	14.103	ABH-SSY2
*Zingiber officinale* Roscoe.	rhizomes	12.821	ABH-SSY3
*Piper nigrum* Linn.	fruits	11.538	ABH-SSY4
*Mesua ferrea* Linn.	flowers	10.256	ABH-SSY5
*Myristica fragrans* Houtt.	fruits	8.974	ABH-SSY6
*Aucklandia lappa* Decne.	roots	7.692	ABH-SSY7
*Nigella sativa* Linn.	seeds	6.410	ABH-SSY8
*Cinnamomum bejolghota* Buch.-Ham.	barks	5.128	ABH-SSY9
*Kleinhovia hospita* Linn.	stem	3.846	ABH-SSY10
*Azadirachta indica* A. Juss.	leaves	2.564	ABH-SSY11
Camphor	-	1.282	-

**Table 2:** The % yield and antioxidant activity of each medicinal herb extract in SSY.

Scientific Name	% Yield (*w/w*)	DPPH Assay IC_50_ (mg/mL)	ABTS Assay IC_50_ (mg/mL)
*Cannabis sativa* Linn.	15.717	1.322 ± 0.012	0.249 ± 0.009
*Piper retrofractum* Vahl.	12.451	0.799 ± 0.010	0.640 ± 0.038
*Zingiber officinale* Roscoe.	4.615	0.285 ± 0.004	0.055 ± 0.005
*Piper nigrum* Linn.	8.362	0.632 ± 0.080	0.224 ± 0.010
*Mesua ferrea* Linn.	17.671	0.057 ± 0.000	0.021 ± 0.001
*Myristica fragrans* Houtt.	19.680	0.563 ± 0.007	0.071 ± 0.002
*Aucklandia lappa* Decne.	22.125	0.598 ± 0.006	0.487 ± 0.022
*Nigella sativa* Linn.	50.448	8.460 ± 0.053	2.047 ± 0.047
*Cinnamomum bejolghota* Buch.-Ham.	14.653	0.011 ± 0.000	0.013 ± 0.000
*Kleinhovia hospita* Linn.	2.443	0.295 ± 0.004	0.093 ± 0.001
*Azadirachta indica* A. Juss.	19.615	0.073 ± 0.001	0.068 ± 0.004
Camphor	-	>1000	>1000
Suk-SaiYasna	18.245	0.682 ± 0.010	0.199 ± 0.004
Trolox (µM)		39.178 ± 0.134	54.968 ± 4.088

**Table 3:** The quantity of chemical constituents in *Cannabis sativa* Linn. leaves and Suk-SaiYasna (SSY) extracts in LC-MS/MS analysis.

Chemical Constituent	*Cannabis sativa* Linn. (mg/g Extract)	Suk-SaiYasna (mg/g Extract)
CBD	4.09 ± 0.05	0.03 ± 0.00
delta-9-THC	5.94 ± 0.04	1.04 ± 0.01
delta-8-THC	n.a.	n.a.
THCA-A	2.44 ± 0.03	0.61 ± 0.01

**Table 4:** The quantity of chemical constituents in the 95% ethanolic extract of the SSY remedy.

Chemical Constituent	Amount of Chemical Constituent (mg/g Extract)	
Piperine	*Piper retrofractum* Vahl.	5.690 ± 0.011
	*Piper nigrum* Linn.	4.375 ± 0.009
	SSY	8.853 ± 0.014
6-Gingerol	*Zingiber officinale* Roscoe.	13.556 ± 0.012
	SSY	1.687 ± 0.003
Gallic acid	*Mesua ferrea* Linn.	175.047 ± 0.344
	*Kleinhovia hospita* Linn.	6.745 ± 0.014
	SSY	167.777 ± 0.079
Myricetin	*Mesua ferrea* Linn.	18.510 ± 0.063
	*Kleinhovia hospita* Linn.	1.247 ± 0.005
	SSY	17.225 ± 0.026
Myristicin	*Myristica fragrans* Houtt.	7.391 ± 0.009
	SSY	6.847 ± 0.009
Thymoquinone	*Nigella sativa* Linn.	15.431 ± 0.048
	SSY	2.262 ± 0.024
Cinnamic acid	*Cinnamomum bejolghota* Buch.-Ham.	3.931 ± 0.008
	SSY	4.662 ± 0.001
Costunolide	*Aucklandia lappa* Decne.	12.522 ± 0.007
	SSY	7.911 ± 0.008
Azadirachtin	*Azadirachta indica* A. Juss.	10.278 ± 0.050
	SSY	2.146 ± 0.073

**Table 5:** Primer sequences.

Gene	Forward Primer (5’-3’)	Reverse Primer (5’-3’)	Reference
Keap1	CATCCACCCTAAGGTCATGGA	GACAGGTTGAGAACTCCTCC	[50]
Nrf2	CAGTGCTCCTATGCGTGAA	GCGGCTTGAATGTTTGTC	[51]
HO-1	ACAGATGGCGTCACTTCG	TGAGGACCCACTGGAGGA	
NQO1	CTTTAGGGTCGTCTTGG	CAATCAGGGCTCTTCTCG	
GAPDH	ACCACAGTCCATGCCATCAC	TCCACCACCCTGTTGCTGTA	[52]

## Data Availability

The data used to support the findings of this study can be made available by the corresponding authors upon request.

## Supplementary Materials

The following supporting information can be downloaded at https://www.mdpi.com/article/10.3390/ijms26115388/s1.

## Abbreviations

The following abbreviations are used in this manuscript: .

SSY	Suk-SaiYasna
UCMS	Unpredictable chronic mild stress
HPA	Hypothalamic-pituitary-adrenal
ROS	Reactive oxygen species
Nrf2	Nuclear factor erythroid 2-related factor 2
Keap1	Kelch-like ECH-associated protein 1
SOD	Superoxide dismutase
CAT	Catalase
ECS	Endocannabinoid system
CBD	Cannabidiol
NQO1	NAD(P)H quinone dehydrogenase 1
HO-1	Heme oxygenase-1
MWM	Morris water maze
NORT	Novel object recognition test
TBARS	Thiobarbituric acid reactive substances
MDA	Malondialdehyde
SCMC	Sodium carboxymethyl cellulose
DPPH	2,2-diphenyl-1-picrylhydrazyl
ABTS	2,2′-azinobis-(3-ethylbenzothiazoline-6-sulfonic acid)
LOD	Limit of detection
LOQ	Limit of quantification
