# Supplementary material for: Suk-SaiYasna Remedy, a Traditional Thai Medicine, Mitigates Stress-Induced Cognitive Impairment via Keap1-Nrf2 Pathway
Source: Int J Mol Sci. 2025 Jun 4;26(11):5388. doi: 10.3390/ijms26115388 (PMC12154420; doi:10.3390/ijms26115388)
Supplement: Supplementary file 1 [file ijms-26-05388-s001.zip › ijms-3626821-supplementary.pdf]

Supplementary Materials
Suk-SaiYasna Remedy, A Traditional Thai Medicine, Mitigates Stress-Induced Cognitive Impairment via Keap1-Nrf2 Pathway

Wuttipong Masraksa [1], Supawadee Daodee [2], Orawan Monthakantirat [2], Chantana Boonyarat [2], Charinya Khamphukdee [3], Pakakrong Kwankhao [4], Abdulwaris Mading [1], Poowanarth Muenhong [2], Juthamart Maneenet [5], Suresh Awale [5], Kinzo Matsumoto [6,7] and Yaowared Chulikhit [2,*]

1 Graduate School of Pharmaceutical Sciences, Khon Kaen University, Khon Kaen 40002, Thailand; wuttipongmas@kkumail.com (W.M.); abdulwaris.m@kkumail.com (A.M.)
2 Division of Pharmaceutical Chemistry, Faculty of Pharmaceutical Sciences, Khon Kaen University, Khon Kaen 40002, Thailand; csupawad@kku.ac.th (S.D.); oramon@kku.ac.th (O.M.); chaboo@kku.ac.th (C.B.); poowme@kku.ac.th (P.M.)
3 Division of Pharmacognosy and Toxicology, Faculty of Pharmaceutical Sciences, Khon Kaen University, Khon Kaen 40002, Thailand; charkh@kku.ac.th
4 Center of Evidence-Based for Traditional and Herbal Medicine, Chao Phya Abhaibhubejhr Hospital, Prachinburi 25000, Thailand; pakakrong2@gmail.com
5 Natural Drug Discovery Laboratory, Institute of Natural Medicine, University of Toyama, 2630 Sugitani, Toyama 930-0194, Japan; juthamart_pp@hotmail.com (J.M.); suresh@inm.u-toyama.ac.jp (S.A.)
6 Graduate School of Pharmaceutical Sciences, Daiichi University of Pharmacy, Fukuoka 815-8511, Japan; k-matsumoto@daiichi-cps.ac.jp
7 Division of Medicinal Pharmacology, Institute of Natural Medicine, University of Toyama, 2630 Sugitani, Toyama 930-0194, Japan
* Correspondence: yaosum@kku.ac.th; Tel.: +66-81-380-2357

1. ***Statistical analysis of SSY on Unpredictable Chronic Mild Stress (UCMS)-Induced Cognitive Impairment.***

Table S1. Pair Student' s *t*-test and One-way analysis of variance (ANOVA) test of escape latency time (training phase session) on Morris Water Maze (MWM) test

Group comparison	ANOVA followed by Tukey's post hoc test	
	P	***F*(DF$_{between\ group}$, DF$_{residual}$)**
**Day 2**		
non-stress group VS. UCMS + vehicle group	< 0.001	*t*(22) = -4.820
UMCS + vehicle group vs. UCMS + Vitamin E group	Not significant	*F*(4,55) = 4.183
UMCS + vehicle group vs. UCMS + SSY20 group	Not significant	
UMCS + vehicle group vs. UCMS + SSY100 group	Not significant	
UMCS + vehicle group vs. UCMS + SSY500 group	Not significant	
**Day 3**		
non-stress group VS. UCMS + vehicle group	< 0.001	*t*(22) = -7.783
UMCS + vehicle group vs. UCMS + Vitamin E group	< 0.001	*F*(4,55) = 13.026
UMCS + vehicle group vs. UCMS + SSY20 group	Not significant	
UMCS + vehicle group vs. UCMS + SSY100 group	< 0.05	
UMCS + vehicle group vs. UCMS + SSY500 group	< 0.001	
**Day 4**		
non-stress group VS. UCMS + vehicle group	< 0.001	*t*(22) = -7.422
UMCS + vehicle group vs. UCMS + Vitamin E group	< 0.001	*F*(4,55) = 21.031
UMCS + vehicle group vs. UCMS + SSY20 group	Not significant	
UMCS + vehicle group vs. UCMS + SSY100 group	< 0.001	
UMCS + vehicle group vs. UCMS + SSY500 group	< 0.001	
**Day 5**		
non-stress group VS. UCMS + vehicle group	< 0.001	*t*(22) = -10.101
UMCS + vehicle group vs. UCMS + Vitamin E group	< 0.001	*F*(4,55) = 12.177
UMCS + vehicle group vs. UCMS + SSY20 group	Not significant	
UMCS + vehicle group vs. UCMS + SSY100 group	< 0.001	
UMCS + vehicle group vs. UCMS + SSY500 group	< 0.001	

Table S2. Pair Student' s *t*-test and One-way analysis of variance (ANOVA) test of time in target quadrant (probe test session) on MWM test

Group comparison	ANOVA followed by Tukey's post hoc test	
	P	***F*(DF$_{between\ group}$, DF$_{residual}$)**
non-stress group VS. UCMS + vehicle group	< 0.001	*t*(22) = 9.745
UMCS + vehicle group vs. UCMS + Vitamin E group	< 0.001	*F*(4,55) = 6.970
UMCS + vehicle group vs. UCMS + SSY20 group	Not significant	
UMCS + vehicle group vs. UCMS + SSY100 group	< 0.05	
UMCS + vehicle group vs. UCMS + SSY500 group	< 0.001	
UCMS + SSY20 group vs. UCMS + SSY500 group	< 0.05	

Table S3. Pair Student' s *t*-test and One-way analysis of variance (ANOVA) test of % spontaneous alternation on Y-maze test

**Group comparison**	**ANOVA followed by Tukey's post hoc test**	
	P	**$F(DF_{between\ group}, DF_{residual})$**
non-stress group VS. UCMS + vehicle group	< 0.001	$t(22) = 10.885$
UMCS + vehicle group vs. UCMS + Vitamin E group	< 0.001	$F(4,55) = 21.045$
UMCS + vehicle group vs. UCMS + SSY20 group	Not significant	
UMCS + vehicle group vs. UCMS + SSY100 group	< 0.001	
UMCS + vehicle group vs. UCMS + SSY500 group	< 0.001	
UCMS + SSY20 group vs. UCMS + SSY100 group	Not significant	
UCMS + SSY20 group vs. UCMS + SSY500 group	< 0.001	
UCMS + SSY100 group vs. UCMS + SSY500 group	0.006	

Table S4. Pair Student' s *t*-test and One-way analysis of variance (ANOVA) test of % discrimination index on Novel Object Recognition Test (NORT)

**Group comparison**	**ANOVA followed by Tukey's post hoc test**	
	P	**$F(DF_{between\ group}, DF_{residual})$**
non-stress group VS. UCMS + vehicle group	< 0.001	$t(22) = 15.180$
UMCS + vehicle group vs. UCMS + Vitamin E group	< 0.001	$F(4,55) = 7.664$
UMCS + vehicle group vs. UCMS + SSY20 group	Not significant	
UMCS + vehicle group vs. UCMS + SSY100 group	< 0.001	
UMCS + vehicle group vs. UCMS + SSY500 group	< 0.001	
UCMS + SSY20 group vs. UCMS + SSY100 group	Not significant	
UCMS + SSY20 group vs. UCMS + SSY500 group	0.048	
UCMS + SSY100 group vs. UCMS + SSY500 group	Not significant	

2. ***Statistical analysis of the SSY extract on UCMS-Induced hypersecretion of lipid peroxidation, Superoxide dismutase (SOD) (B) and Catalase (CAT)***

Table S5. Pair Student' s *t*-test and One-way analysis of variance (ANOVA) test of concentration of MDA (nmol/mg protein) in lipid peroxidation test

**Frontal cortex**		
**Group comparison**	**ANOVA followed by Tukey's post hoc test**	
	P	**$F(DF_{between\ group}, DF_{residual})$**
non-stress group VS. UCMS + vehicle group	< 0.001	$t(8) = -6.102$
UMCS + vehicle group vs. UCMS + Vitamin E group	< 0.001	
UMCS + vehicle group vs. UCMS + SSY20 group	Not significant	
UMCS + vehicle group vs. UCMS + SSY100 group	0.006	
UMCS + vehicle group vs. UCMS + SSY500 group	< 0.001	$F(4,20) = 5.162$
UCMS + SSY20 group vs. UCMS + SSY100 group	Not significant	
UCMS + SSY20 group vs. UCMS + SSY500 group	0.040	
UCMS + SSY100 group vs. UCMS + SSY500 group	Not significant	
**Hippocampus**		
non-stress group VS. UCMS + vehicle group	< 0.001	$t(8) = -4.832$
UMCS + vehicle group vs. UCMS + Vitamin E group	< 0.001	
UMCS + vehicle group vs. UCMS + SSY20 group	Not significant	
UMCS + vehicle group vs. UCMS + SSY100 group	0.038	
UMCS + vehicle group vs. UCMS + SSY500 group	< 0.001	$F(4,20) = 3.376$
UCMS + SSY20 group vs. UCMS + SSY100 group	Not significant	
UCMS + SSY20 group vs. UCMS + SSY500 group	0.033	
UCMS + SSY100 group vs. UCMS + SSY500 group	Not significant	

Table S6. Pair Student' s *t*-test and One-way analysis of variance (ANOVA) test of SOD activity (U/mg protein) in SOD test

**Frontal cortex**		
**Group comparison**	**ANOVA followed by Tukey's post hoc test**	
	P	**$F(DF_{between\ group}, DF_{residual})$**
non-stress group VS. UCMS + vehicle group	< 0.001	$t(8) = 7.875$
UMCS + vehicle group vs. UCMS + Vitamin E group	< 0.001	
UMCS + vehicle group vs. UCMS + SSY20 group	Not significant	
UMCS + vehicle group vs. UCMS + SSY100 group	0.048	
UMCS + vehicle group vs. UCMS + SSY500 group	< 0.001	$F(4,20) = 18.332$
UCMS + SSY20 group vs. UCMS + SSY100 group	0.034	
UCMS + SSY20 group vs. UCMS + SSY500 group	0.005	
UCMS + SSY100 group vs. UCMS + SSY500 group	Not significant	
**Hippocampus**		
non-stress group VS. UCMS + vehicle group	< 0.001	$t(8) = 8.279$
UMCS + vehicle group vs. UCMS + Vitamin E group	< 0.001	
UMCS + vehicle group vs. UCMS + SSY20 group	Not significant	
UMCS + vehicle group vs. UCMS + SSY100 group	0.021	
UMCS + vehicle group vs. UCMS + SSY500 group	< 0.001	$F(4,20) = 21.167$
UCMS + SSY20 group vs. UCMS + SSY100 group	Not significant	
UCMS + SSY20 group vs. UCMS + SSY500 group	0.029	
UCMS + SSY100 group vs. UCMS + SSY500 group	Not significant	

Table S7. Pair Student' s *t*-test and One-way analysis of variance (ANOVA) test of CAT activity (U/mg protein) in CAT test

Frontal cortex		
**Group comparison**	**ANOVA followed by Tukey's post hoc test**	
	P	***F*(DF$_{between\ group}$, DF$_{residual}$)**
non-stress group VS. UCMS + vehicle group	< 0.001	$t(8) = 7.879$
UMCS + vehicle group vs. UCMS + Vitamin E group	< 0.001	$F(4,20) = 46.629$
UMCS + vehicle group vs. UCMS + SSY20 group	Not significant	
UMCS + vehicle group vs. UCMS + SSY100 group	< 0.001	
UMCS + vehicle group vs. UCMS + SSY500 group	< 0.001	
UCMS + SSY100 group vs. UCMS + SSY500 group	0.036	
UCMS + SSY20 group vs. UCMS + SSY500 group	< 0.001	
UCMS + SSY20 group vs. UCMS + SSY100 group	Not significant	
**Hippocampus**		
non-stress group VS. UCMS + vehicle group	< 0.001	$t(8) = 6.860$
UMCS + vehicle group vs. UCMS + Vitamin E group	< 0.001	$F(4,20) = 52.970$
UMCS + vehicle group vs. UCMS + SSY20 group	Not significant	
UMCS + vehicle group vs. UCMS + SSY100 group	< 0.001	
UMCS + vehicle group vs. UCMS + SSY500 group	< 0.001	
UCMS + SSY100 group vs. UCMS + SSY500 group	0.045	
UCMS + SSY20 group vs. UCMS + SSY500 group	< 0.001	
UCMS + SSY20 group vs. UCMS + SSY100 group	Not significant	

3. ***Statistical Analysis of Effect of the SSY on UCMS-Induced Impaired Antioxidant enzyme Frontal Cortex and Hippocampus Using Quantitative real-time polymerase chain reaction (qPCR)***

Table S8. Paired Student's *t*-test and One-way analysis of variance (ANOVA) test of Keap1 in frontal cortex and hippocampus

Frontal cortex		
**Group comparison**	**ANOVA followed by Tukey's post hoc test**	
	P	***F*(DF$_{between\ group}$, DF$_{residual}$)**
non-stress group VS. UCMS + vehicle group	< 0.001	$t(8) = -25.280$
UMCS + vehicle group vs. UCMS + Vitamin E group	< 0.001	$F(4,20) = 37.125$
UMCS + vehicle group vs. UCMS + SSY20 group	Not significant	
UMCS + vehicle group vs. UCMS + SSY100 group	< 0.001	
UMCS + vehicle group vs. UCMS + SSY500 group	< 0.001	
UCMS + SSY100 group vs. UCMS + SSY500 group	0.021	
UCMS + SSY20 group vs. UCMS + SSY500 group	< 0.001	
UCMS + SSY20 group vs. UCMS + SSY100 group	0.001	
**Hippocampus**		
non-stress group VS. UCMS + vehicle group	< 0.001	$t(8) = -22.411$
UMCS + vehicle group vs. UCMS + Vitamin E group	< 0.001	$F(4,20) = 107.041$
UMCS + vehicle group vs. UCMS + SSY20 group	Not significant	
UMCS + vehicle group vs. UCMS + SSY100 group	< 0.001	
UMCS + vehicle group vs. UCMS + SSY500 group	< 0.001	
UCMS + SSY100 group vs. UCMS + SSY500 group	0.041	
UCMS + SSY20 group vs. UCMS + SSY500 group	< 0.001	
UCMS + SSY20 group vs. UCMS + SSY500 group	0.002	

Table S9. Paired Student's *t*-test and One-way analysis of variance (ANOVA) test of nuclear factor erythroid 2-related factor 2 (Nrf2) in frontal cortex and hippocampus

**Frontal cortex**		
**Group comparison**	**ANOVA followed by Tukey's post hoc test**	
	P	**$F(DF_{between\ group}, DF_{residual})$**
non-stress group VS. UCMS + vehicle group	< 0.001	$t(8) = 55.337$
UMCS + vehicle group vs. UCMS + Vitamin E group	< 0.001	$F(4,20) = 50.343$
UMCS + vehicle group vs. UCMS + SSY20 group	Not significant	
UMCS + vehicle group vs. UCMS + SSY100 group	< 0.001	
UMCS + vehicle group vs. UCMS + SSY500 group	< 0.001	
UCMS + SSY20 group vs. UCMS + SSY500 group	0.039	
UCMS + SSY100 group vs. UCMS + SSY500 group	Not significant	
UCMS + SSY20 group vs. UCMS + SSY100 group	0.011	
**Hippocampus**		
non-stress group VS. UCMS + vehicle group	< 0.001	$t(8) = 35.777$
UMCS + vehicle group vs. UCMS + Vitamin E group	< 0.001	$F(4,20) = 70.092$
UMCS + vehicle group vs. UCMS + SSY20 group	Not significant	
UMCS + vehicle group vs. UCMS + SSY100 group	< 0.001	
UMCS + vehicle group vs. UCMS + SSY500 group	< 0.001	
UCMS + SSY20 group vs. UCMS + SSY500 group	0.037	
UCMS + SSY20 group vs. UCMS + SSY500 group	Not significant	
UCMS + SSY20 group vs. UCMS + SSY100 group	Not significant	

Table S10. Paired Student's *t*-test and One-way analysis of variance (ANOVA) test of heme oxygenase-1 (HO-1) in frontal cortex and hippocampus

**Frontal cortex**		
**Group comparison**	**ANOVA followed by Tukey's post hoc test**	
	P	**$F(DF_{between\ group}, DF_{residual})$**
non-stress group VS. UCMS + vehicle group	< 0.001	$t(8) = 13.809$
UMCS + vehicle group vs. UCMS + Vitamin E group	< 0.001	$F(4,20) = 51.588$
UMCS + vehicle group vs. UCMS + SSY20 group	Not significant	
UMCS + vehicle group vs. UCMS + SSY100 group	< 0.001	
UMCS + vehicle group vs. UCMS + SSY500 group	< 0.001	
UCMS + SSY20 group vs. UCMS + SSY500 group	< 0.001	
UCMS + SSY100 group vs. UCMS + SSY500 group	< 0.001	
**Hippocampus**		
non-stress group VS. UCMS + vehicle group	< 0.001	$t(8) = 45.882$
UMCS + vehicle group vs. UCMS + Vitamin E group	< 0.001	$F(4,20) = 54.644$
UMCS + vehicle group vs. UCMS + SSY20 group	Not significant	
UMCS + vehicle group vs. UCMS + SSY100 group	< 0.001	
UMCS + vehicle group vs. UCMS + SSY500 group	< 0.001	
UCMS + SSY100 group vs. UCMS + SSY500 group	< 0.001	
UCMS + SSY20 group vs. UCMS + SSY100 group	< 0.001	
UCMS + SSY20 group vs. UCMS + SSY500 group	< 0.001	

Table S11. Paired Student's *t*-test and One-way analysis of variance (ANOVA) test of NAD(P)H quinone dehydrogenase 1 (NQO1) in frontal cortex and hippocampus

Frontal cortex		
**Group comparison**	**ANOVA followed by Tukey's post hoc test**	
	P	**$F(DF_{between\ group}, DF_{residual})$**
non-stress group VS. UCMS + vehicle group	< 0.001	*t*(8) = -10.176
UMCS + vehicle group vs. UCMS + Vitamin E group	< 0.001	*F*(4,20) = 95.769
UMCS + vehicle group vs. UCMS + SSY20 group	Not significant	
UMCS + vehicle group vs. UCMS + SSY100 group	< 0.001	
UMCS + vehicle group vs. UCMS + SSY500 group	< 0.001	
UMCS + SSY20 group vs. UCMS + SSY100 group	Not significant	
UCMS + SSY20 group vs. UCMS + SSY500 group	< 0.001	
UCMS + SSY100 group vs. UCMS + SSY500 group	< 0.001	
**Hippocampus**		
non-stress group VS. UCMS + vehicle group	< 0.001	*t*(8) = -17.656
UMCS + vehicle group vs. UCMS + Vitamin E group	< 0.001	*F*(4,20) = 56.235
UMCS + vehicle group vs. UCMS + SSY20 group	Not significant	
UMCS + vehicle group vs. UCMS + SSY100 group	< 0.001	
UMCS + vehicle group vs. UCMS + SSY500 group	< 0.001	
UMCS + SSY20 group vs. UCMS + SSY100 group	0.026	
UMCS + SSY100 group vs. UCMS + SSY500 group	< 0.001	
UMCS + SSY20 group vs. UCMS + SSY500 group	< 0.001	

Table S12. Validation results of the LC-MS/MS method.

Chemical constituent	Retention time (min)	ION precursor/product	MS Mode	Standard curve equations	R^2	LOD (μg/mg)	LOQ (μg/mg)
**CBD**	10.50	315.3/193.197, 259.238	ESI positive	y = 737.57x + 17.350	0.9960	0.03	0.11
**delta-9-THC**	12.10	315.3/193.197, 259.238	ESI positive	y = 1101.7x + 145.90	0.9920	0.07	0.22
**delta-8-THC**	12.15	315.3/193.197, 259.238	ESI positive	y = 816.87x + 69.338	0.9932	0.04	0.12
**THCA-A**	12.75	357.2/313.28, 245.137	ESI negative	y = 71.227x + 29.183	0.9949	0.15	0.49

Table S13. Validation results of the analytical method for determination of piperine, gingerol, gallic acid, myricetin, myristicin, thymoquinone, cinnamic acid, costunolide, and azadirachtin content in the SSY extract

**Chemical constituent**	**Linearity**	**Range (μg/ml)**	**Accuracy (%Recovery)**	**Precision Repeatability (%RSD)**	**Precision Intermediate Precision (%RSD)**	**LOD (S/N)**	**LOQ (S/N)**	**UV Purity**	**Robustness (Flowrate) (%RSD)**
Piperine	y = 272.52x - 110.36 R^2 = 0.9960	1-8	95.55-98.33	0.04-0.54	0.05-1.11	0.1 (5.98)	0.2 (12.70)	999.96	0.4, 0.5, 0.6 (0.64-2.77)
6-Gingerol	y = 134.14x - 43.639 R^2 = 0.9988	1-10	96.34-98.09	0.05-1.33	0.03-1.20	0.1 (14.97)	0.2 (20.78)	999.94	0.4, 0.5, 0.6 (0.22-0.58)
Gallic acid	y = 134.14x - 43.639 R^2 = 0.9988	1-8	98.45-100.91	0.04-0.44	0.01-0.04	0.1 (20.34)	0.2 (20.36)	996.46	0.4, 0.5, 0.6 (0.16-0.18)
Myricetin	y = 97.20x - 12.502 R^2 = 0.9981	1-8	100.51-103.50	0.23-0.83	0.03-0.19	0.1 (17.30)	0.2 (13.05)	996.57	0.4, 0.5, 0.6 (0.08-0.52)
Myristicin	y = 419.38x – 134.70 R^2 = 0.999	1-10	98.10-104.56	0.04-0.64	0.04-0.65	0.1 (37.89)	0.2 (14.51)	999.84	0.4, 0.5, 0.6 (0.04-0.05)
Thymoquinone	y = 29.565x + 1.627 R^2 = 0.9977	1-10	99.81-101.55	0.30-0.77	0.02-1.20	0.1 (33.06)	0.2 (12.67)	999.99	0.4, 0.5, 0.6 (0.07-3.06)
Cinnamic acid	y = 266.83x + 63.244 R^2 = 0.9974	1-10	95.37-103.77	0.12-0.90	0.11-0.92	0.1 (21.96)	0.2 (28.24)	999.93	0.4, 0.5, 0.6 (0.20-0.30)
Costunolide	y = 161.74x + 5.0821 R^2 = 0.9967	1-10	90.04-103.05	0.16-0.84	0.07-0.87	0.1 (20.71)	0.2 (70.42)	999.89	0.4, 0.5, 0.6 (0.61-0.63)
Azadirachtin	y = 24.738x + 2.7889 R^2 = 0.9998	1-10	99.03-100.51	0.23-1.02	0.02-1.50	0.1 (34.59)	0.2 (15.39)	999.94	0.4, 0.5, 0.6 (0.43-0.39)
**Acceptance criteria**	R^2 = 0.99	-	80-115	%RSD ≤ 6	%RSD ≤ 6	S/N ≥ 3	S/N ≥ 10	-	%RSD ≤ 6

Figure S1. The linearity of y-axis representing Area (mAU*s) and the x-axis indicating concentration (microg/ml) for all reference standards

The y-axis value is Area (mAU*s)
The x-axis is concentration (microg/ml).

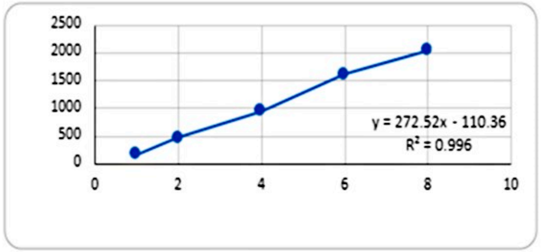

Piperine

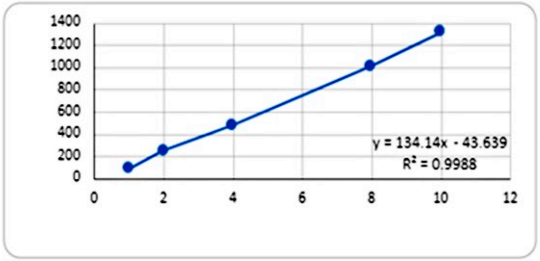

6-Gingerol

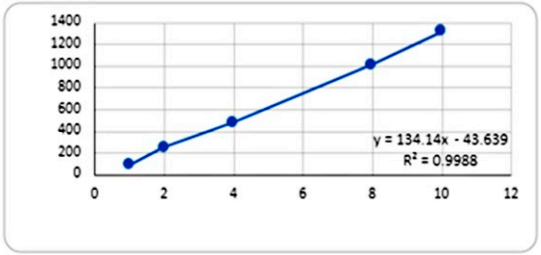

Gallic acid

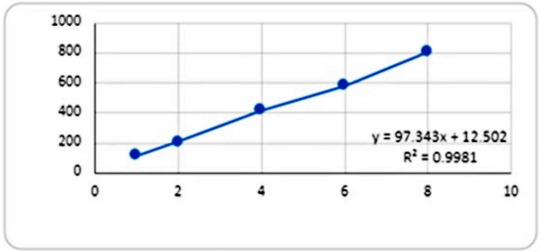

Myricetin

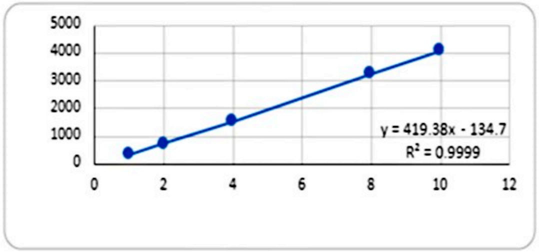

Myristicin

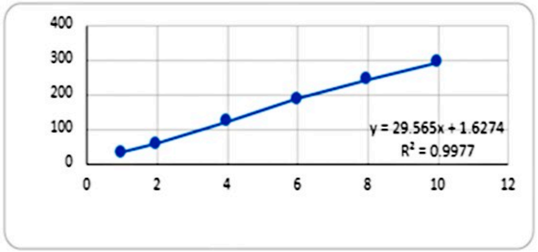

Thymoquinone

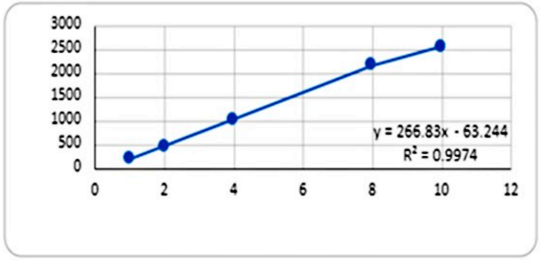

Cinnamic acid

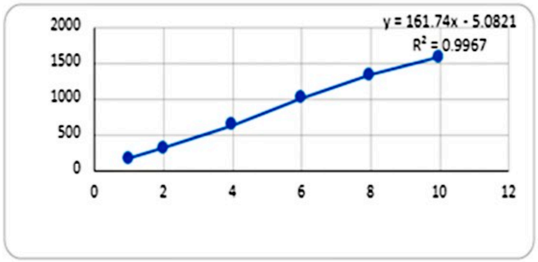

Costunolide

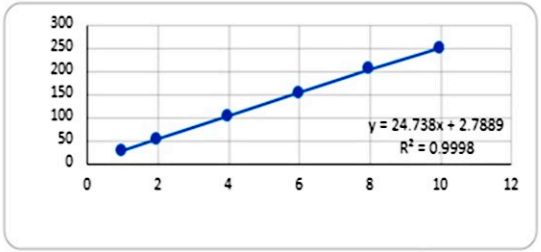

Azadirachtin

Table S14. One-week schedule of unpredictable chronic mild stresses protocol.

Activity/Days	Mon.	Tue.	Wed.	Thu.	Fri.	Sat.	Sun.
1. **Deprivation of food and water (18 hrs.)**	3:00 pm-9:00 am						
2. **Sucrose preference test (1 hr.)**		9:00 am-3:00 pm					
3. **Tilted cage 45° (12, 12 hrs.)**		08:00 pm-08:00 am				7:00 pm-7.00 am	
4. **5 micro pellets restricted access to food (1 hr.)**		3:00-4:00 pm					
5. **Empty bottle exposure (3, 3 hrs.)**			2:00 pm-5:00 pm		9:00 am-12:00 am		
6. **Wet cage 100 g sawdust bedding (21 hrs.)**					1:00 pm-10:00 am		
7. **exposure to light (36 hrs.)**	(Sun.) 6:00 am-(Mon.) 6:00 pm		7:00 pm-07:00 am				(Sun.) 6:00 am-(Mon.) -6:00 pm
8. **Sound is intermittent (3, 5 hrs.)**			9:00 am-12:00 pm			12:00 pm-5:00 am	
9. **Caging in pairs (2 hrs.)**		5:00 pm-7:00 pm					1:00 pm-3:00 pm
